# Amide proton transfer weighted and diffusion weighted imaging based radiomics classification algorithm for predicting 1p/19q co-deletion status in low grade gliomas

**DOI:** 10.1186/s12880-024-01262-z

**Published:** 2024-04-10

**Authors:** Andong Ma, Xinran Yan, Yaoming Qu, Haitao Wen, Xia Zou, Xinzi Liu, Mingjun Lu, Jianhua Mo, Zhibo Wen

**Affiliations:** Department of Radiology, Zhujiang Hospital, Southern Medical University, Haizhu District, 253 Gongye Middle Avenue, Guangzhou, Guangdong 510282 China

**Keywords:** Low-grade glioma, 1p/19q Co-deletion, Prediction, Radiomics, Magnetic resonance imaging, Amide proton transfer weighted imaging, Diffusion weighted imaging

## Abstract

**Background:**

1p/19q co-deletion in low-grade gliomas (LGG, World Health Organization grade II and III) is of great significance in clinical decision making. We aim to use radiomics analysis to predict 1p/19q co-deletion in LGG based on amide proton transfer weighted (APTw), diffusion weighted imaging (DWI), and conventional MRI.

**Methods:**

This retrospective study included 90 patients histopathologically diagnosed with LGG. We performed a radiomics analysis by extracting 8454 MRI-based features form APTw, DWI and conventional MR images and applied a least absolute shrinkage and selection operator (LASSO) algorithm to select radiomics signature. A radiomics score (Rad-score) was generated using a linear combination of the values of the selected features weighted for each of the patients. Three neuroradiologists, including one experienced neuroradiologist and two resident physicians, independently evaluated the MR features of LGG and provided predictions on whether the tumor had 1p/19q co-deletion or 1p/19q intact status. A clinical model was then constructed based on the significant variables identified in this analysis. A combined model incorporating both the Rad-score and clinical factors was also constructed. The predictive performance was validated by receiver operating characteristic curve analysis, DeLong analysis and decision curve analysis. *P* < 0.05 was statistically significant.

**Results:**

The radiomics model and the combined model both exhibited excellent performance on both the training and test sets, achieving areas under the curve (AUCs) of 0.948 and 0.966, as well as 0.909 and 0.896, respectively. These results surpassed the performance of the clinical model, which achieved AUCs of 0.760 and 0.766 on the training and test sets, respectively. After performing Delong analysis, the clinical model did not significantly differ in predictive performance from three neuroradiologists. In the training set, both the radiomic and combined models performed better than all neuroradiologists. In the test set, the models exhibited higher AUCs than the neuroradiologists, with the radiomics model significantly outperforming resident physicians B and C, but not differing significantly from experienced neuroradiologist.

**Conclusions:**

Our results suggest that our algorithm can noninvasively predict the 1p/19q co-deletion status of LGG. The predictive performance of radiomics model was comparable to that of experienced neuroradiologist, significantly outperforming the diagnostic accuracy of resident physicians, thereby offering the potential to facilitate non-invasive 1p/19q co-deletion prediction of LGG.

**Supplementary Information:**

The online version contains supplementary material available at 10.1186/s12880-024-01262-z.

## Introduction

Low-grade gliomas (LGGs) are invasive neoplasms that arise in the cerebral hemispheres of adults including diffuse low-grade and intermediate-grade gliomas (World Health Organization [WHO] grades II and III) [1]. The fifth edition of the WHO Classification of Tumors of the Central Nervous System divides adult-type gliomas into three subtypes based on molecular markers: (1) astrocytoma, isocitrate dehydrogenase (IDH) -mutant, (2) oligodendroglioma, IDH-mutant, and 1p/19q-codeleted, and (3) glioblastoma, IDH-wildtype [2].

Studies have shown that oligodendroglioma has the best prognosis between these three categories [3]. In addition, studies have illustrated that even small residual tumor has a negative impact on overall survival in 1p/19q intact astrocytoma than on 1p/19q co-deleted oligodendroglioma [4]. Therefore, the noninvasive assessment of the molecular subtype of 1p/19q is particularly valuable in guiding clinical decision making.

The radiological features of 1p/19q co-deleted tumors frequently display calcifications, and they predominantly occur in the frontal lobe, with a tendency to invade the gray matter. These tumors typically exhibit heterogeneous signal intensities on both T1- and T2-weighted MR imaging, often lacking a distinct tumor margin [5, 6].

With artificial intelligence technique applied to MRI, radiogenomics becoming a promising tool for discriminating genotype of gliomas in a non-invasive fashion. The largest amount of literature researches focused on conventional MRI, such as T1-weighted imaging (T1WI), T2 weighted imaging (T2WI) and fluid-attenuated inversion recovery (FLAIR) [7–9]. Advanced MRI techniques such as amide proton transfer weighted (APTw) or diffusion weighted imaging (DWI) remain less studied.

APTw imaging is one of the most developed branch of chemical exchange saturation transfer (CEST) imaging [10, 11]. Previous studies have demonstrated that APTw imaging has important value in detecting molecular biomarkers in gliomas, such as IDH mutation, O6-methylguanine methyltransferase (MGMT), and Lys-27-Met mutations in histone 3 genes (H3K27M) [12–16]. In the study by Su et al. [17], CEST imaging was used to identify 1p/19q co-deletion, and statistically significant indices included direct saturation of water (DSW), semi-solid magnetization transfer contrast (MTC), and MTRasym (2.0 ppm). However, the APT value was not statistically significant. In addition, a growing number of researches have shown that features extracted from DWI have predictive values in predicting of glioma molecular subtypes [18–20].

The aim of this retrospective study was to develop a radiogenomics method to predict 1p/19q co-deletion of LGG based on advanced and conventional MRI features.

## Materials and methods

### Patients

**Fig. 1:**
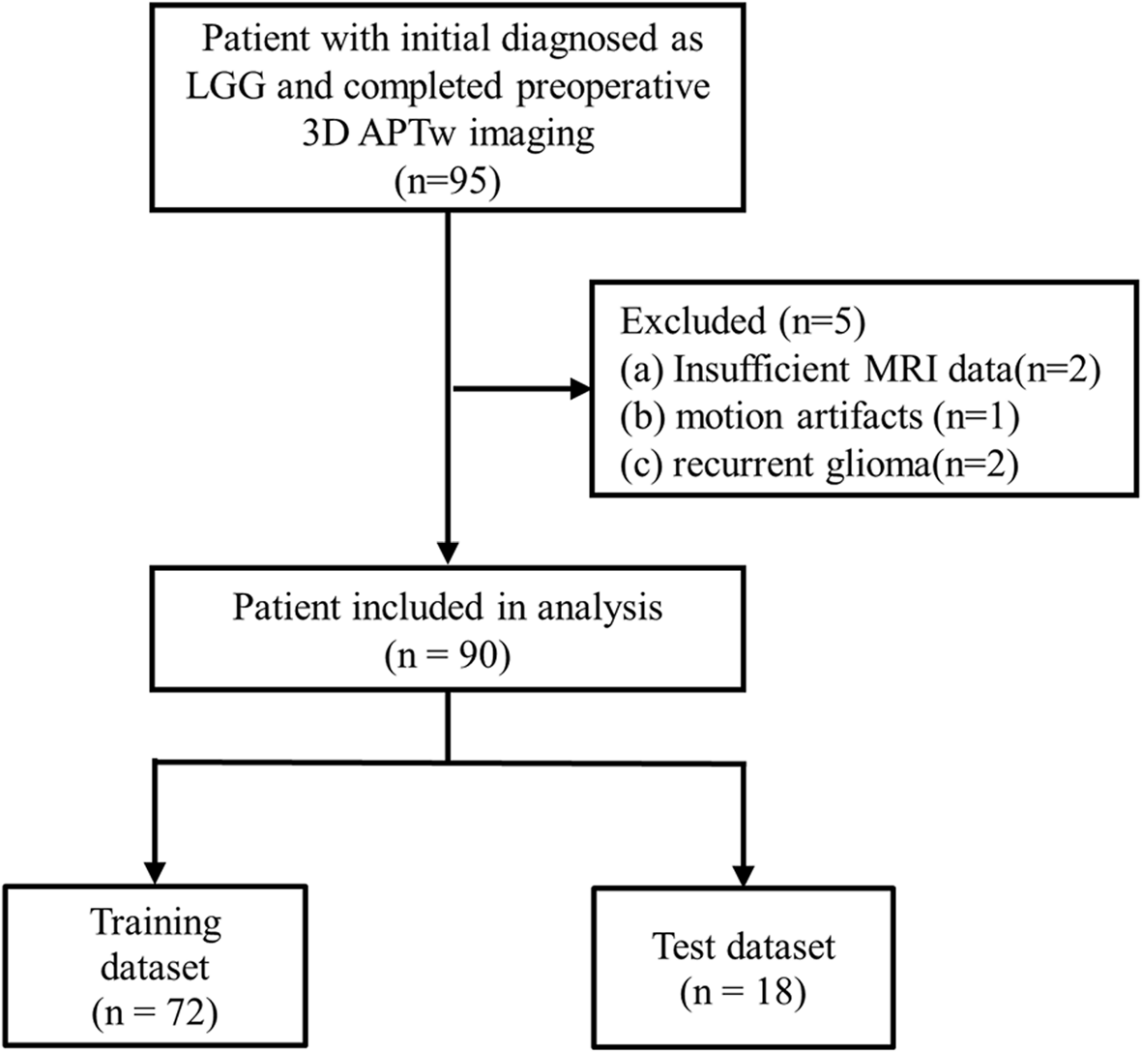
Flowchart of the study population

This study was approved by The Ethics Committee of the Zhujiang Hospital of Southern Medical University, and because its nature of retrospective study, the requirement of obtaining informed consent was waived. We retrospectively analyzed all patients from July 2017 to January 2023, with pathologically diagnosed LGG according to the WHO 2016 Classification and completed preoperative 3D APTw imaging evaluation. A total of 95 patients with initial diagnosed as LGG were reviewed (Fig. 1). The inclusion criteria were as follows: (1) LGG with histopathological confirmation and known 1p/19q co-deletion status; (2) LGG with preoperative MRI including APTw, DWI and corresponding apparent diffusion coefficient (ADC) maps; (3) LGG without any previous treatment at initial diagnosis, and (4) patients over 18 years old. Cases with insufficient MRI data (*n* = 2), MRI data had intense motion artifacts (*n* = 1), patients with recurrent glioma (*n* = 2) were excluded from the study, rendering 90 LGGs in the dataset.

### Evaluation of 1p/19q co-deletion status

1p/19q co-deletion was assessed by a fluorescence in situ hybridization (FISH) locus specific identifier (LSI) probe sets 1p36/1q21 and 19q13/19p13. The assessment was consensus-classified by two pathologists over 6 and 20 years of experience, respectively.

### MRI acquisition

All patients were examined on two 3.0 T Philips scanners (Ingenia Elition 3.0 T X and Ingenia 3.0 T; Philips Medical Systems, Best, The Netherlands) with a 20-channel head-neck coil. In addition to conventional anatomic sequences, each MRI consists of 3D APTw, DWI and the corresponding ADC maps. All image processing and reconstruction algorithms were automatically implemented on MRI scanning system. Among them, the APT sequence encompasses two sets of images: one set is APTw images, and the other set is S0 images. Both sets of images possess the identical spatial resolution. The S0 images represent the control signal intensity without saturation, and the imaging is performed at a frequency offset of − 1560 ppm [11]. Detailed information on imaging parameters are available in the Supplementary Table 1, Additional File 1.

### APTw and DWI imaging parameters

APTW imaging was implemented with a fat-suppressed, mDIXON 3D turbo spin-echo sequence, with RF saturation powers of 2μT and a saturation duration of 2 s were used [11]. The detailed parameters were as follows: SENSE factor, 1.4; repetition time /echo time (TR/TE) = 5900/8 ms; field of view (FOV) = 212 × 182 mm^2^; slice thickness = 5.4 mm; matrix = 120 × 102 (reconstructed to 224 × 224); and voxel size = 1.80 × 1.80 × 5.40 mm^3^ (0.95 × 0.95 × 5.40 mm^3^, reconstructed). A multi-offset, multi-acquisition APTw imaging acquisition protocol was used [7 offsets =  ± 2.7, − 3.5, + 3.5 (3), ± 4.28, − 1560 ppm; value in parentheses is the number of acquisitions, which was considered as one, if not specified]. The total scan time was 4 min 48 s.

DWI was implemented with a 2D single-shot echo-planar imaging sequence. The detailed parameters were as follows: SENSE factor, 2; TR/TE = 3284/200 ms; field of view = 230 × 230 mm^2^; slice thickness = 4.4 mm; matrix = 152 × 122 (reconstructed to 256 × 256); and voxel size = 1.50 × 1.89 × 4.40 mm^3^ (0.90 × 0.90 × 4.40 mm^3^, reconstructed). The ADC maps were calculated using b values of 0 and 1000 s/mm^2^ images.

### MRI feature evaluation

Three neuroradiologists, labeled as Reader A (an experienced neuroradiologist with 7 years of experience) and, Reader B and C (both being resident physicians with 3 and 1 years of experience in neuroradiology respectively) independently evaluated the MR images while blinded to the pathology results. In cases of disagreement, a consensus was reached. The evaluation encompassed the following aspects: gray matter involvement, calcification, hemorrhage, tumor margin clarity (indistinct vs. sharp), and contrast enhancement. Due to limitations in data availability, contrast-enhanced T1-weighted imaging (T1C) was not incorporated into this study. However, where such data were available, we did analyze whether lesions exhibited enhancement.

For each tumor, the readers were also asked to assess whether they believed it exhibited 1p/19q codeletion or was intact, providing a confidence score ranging from 1 (indicating very unsure) to 5 (indicating very sure). This confidence score was then transformed into a prediction "score" by dividing it by 5 and multiplying the result by 1 if the predicted label was 1p/19q codeleted, or by -1 if the predicted label was 1p/19q intact. This approach allowed for the calculation of an Area Under the Curve (AUC) for the manual classification [21].

### Image preprocessing and tumor segmentation

A rigid co-registration was conducted between T1, T2, FLAIR, APT DWI images, and ADC map using SPM12 (https://www.fil.ion.ucl.ac.uk/spm/software/spm12/). The reference volume for coregistration was the unsaturated images (S0 image). Three-dimensional volume of interest (VOI) of whole-tumor were delineated by consensus between two neuroradiologists (reader A and reader B with 7 and 3 years of experience in neuroradiology), blinded to 1p/19q status, using the ITK-SNAP software (http://www.itksnap.org/pmwiki/pmwiki.php). Necrosis, cystic cavities, large vessels, calcification and hemorrhagic components were excluded.

### Extraction of radiomic features

**Fig. 2:**
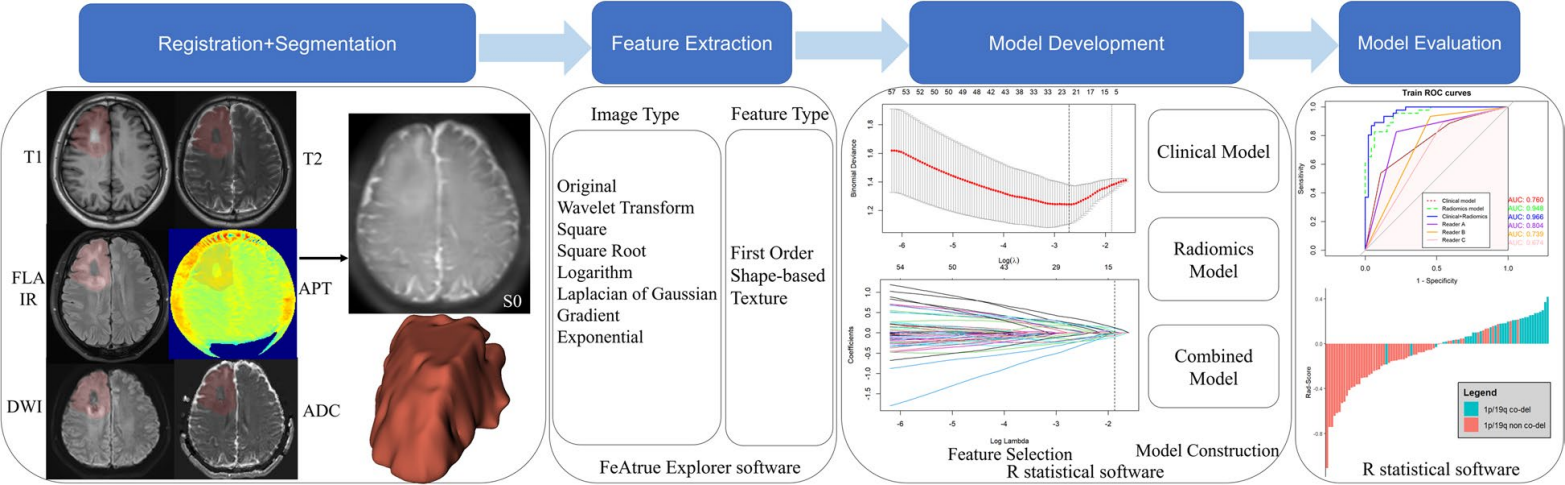
Workflow of the study

All radiomic features were extracted using an open-source software package named FeAture Explorer software (version 0.5.5 https://github.com/salan668/FAE), which was built based on the PyRadiomics package (https://github.com/Radiomics/pyradiomics) [22, 23]. Features were extracted on original image and preprocessed imaging, including wavelet transform, square, square root, logarithm, laplacian of gaussian, gradient and exponential. There are 7 feature types: shape features, first-order features, gray level cooccurrence matrix (GLCM) features, gray level run length matrix (GLRLM) features, gray level size zone matrix (GLSZM) features, neighboring gray tone difference matrix (NGTDM) features, and gray level dependence matrix (GLDM). A total of 8454 features were extracted from the MRI data and 1409 features each from T1WI, T2WI, FLAIR, APTw, DWI and ADC map. A detailed information of the workflow is presented in the Fig. 2.

### Feature selection

The training and test datasets were randomly selected from the dataset at a ratio of 8:2, where the clinical characteristics in the two datasets were balanced. Standardization of radiomic features was performed using z-score intensity normalization and the upsampling was employed to remove the unbalance of the training dataset. Then, a least absolute shrinkage and selection operator (LASSO) algorithm was employed in combination with fivefold cross-validation, which was aimed to identify the best features subset via the one-standard error of the minimum criteria.

### Model development

A total of 3 models were built. Demographic factors and MRI features of the training set were compared between patients with 1p/19q codeleted and those with 1p/19q intact status using multivariable logistic regression analysis. The significant variables identified in this analysis were then used to build a clinical model. Radiomic model was established by using the final selected radiomics features, and a radiomics score (Rad-score) was generated using a linear combination of the values of the selected features weighted for each patient. A combined model was established by employing logistic regression analysis with the significant variables previously identified, along with the Rad-score of the patient.

### Model evaluation

The receiver operating characteristic (ROC) curves and area under the curve (AUC), sensitivity, specificity, positive predictive value (PPV), negative predictive value (NPV) and accuracy were calculated for all models. We used Delong's test to compare the prediction performance of the three models against the individual readers (Reader A, B, and C). A *p* < 0.05 was considered significant. Decision curve analysis (DCA) was conducted to evaluate the clinical consequences of three models by plotting their net benefits across different threshold probabilities [24].

### Statistical analysis

SPSS v.26.0 (IBM SPSS Statistic Version19, Chicago, IL, USA) and R statistical software (v.4.2.2; https://www.r-project.org) were used for statistical analysis. We used independent samples t-test for quantitative data, and Wilcoxon test, chi-square test and Fisher’s exact test for qualitative data. A two-sided *p*-value of < 0.05 was considered significant.

## Results

### Clinical characteristics of patients

The characteristics of the patients in both the training and test datasets are outlined in Table 1. Within the training dataset, the variables of IDH1 status, gray matter involvement, calcification, and tumor margin exhibited statistically significant differences between the patients with 1p/19q codeletion and those with intact 1p/19q status. However, upon performing a multiple logistic regression analysis, only calcification and tumor margin clarity were identified as independent predictors in the clinical model. When evaluating the enhancement of lesion, after excluding four cases without T1C, there was no statistically significant difference in the enhancement patterns of tumors between the 1p/19q co-deleted and 1p/19q intact groups among the remaining 86 cases (*p* = 0.941). Among these cases, 44 (51.2%) cases showed no evidence of enhancement.

**Table 1:** Clinical characteristics of the patients

Clinical factors	Training set (*n* = 72)			Test set (*n* = 18)			*p* value
	1p/19q co-del	1p/19q non co-del	*P* value	1p/19q co-del	1p/19q non co-del	*p* value	
Number	26 (36.1)	46 (63.9)	-	7 (38.9)	11(61.1)	-	0.827
Age(years)^a^	42 (34–46)	43 (33–51)	0.560	52 (41–63)	43(33–59)	0.303	0.081
Sex			0.100			1.000	0.164
Male	13 (50.0)	32 (69.6)		3 (42.9)	5 (45.5)		
Female	13 (50.0)	14 (30.4)		4 (57.1)	6 (54.5)		
WHO grades			0.903			1.000	0.642
Grade II	19 (73.1)	33 (71.7)		5 (71.4)	7 (63.6)		
Grade III	7 (26.9)	13 (28.3)		2 (28.6)	4 (36.4)		
Histology							0.826
Oligodendroglioma	26			6			
Astrocytoma	46			12			
IDH			< 0.001			0.316	0.642
Mutation	25(96.2)	27(58.7)		6(85.7)	6(54.5)		
Wildtype	1(3.8)	19(41.3)		1(14.3)	5(45.5)		
Gray matter involvement			0.003			0.637	0.039
Absent	0(0)	12(26.1)		2(28.6)	5(45.5)		
Present	26(100)	34(73.9)		5(71.4)	6(54.5)		
Calcification			< 0.001			0.141	0.743
Absent	9(34.6)	36(78.3)		3(42.9)	9(81.8)		
Present	17(65.4)	10(21.7)		4(57.1)	2(18.2)		
Hemorrhage			1.000			1.000	0.316
Absent	23(88.5)	40(87.0)		5(71.4)	9(81.8)		
Present	3(11.5)	6(13.0)		2(28.6)	2(18.2)		
Tumor margin			0.009			0.316	0.820
Indistinct	23(88.5)	27 (58.7)		6(85.7)	6(54.5)		
Sharp	3(11.5)	19(41.3)		1(14.3)	5(45.5)		
Contrast enhancement			0.357			0.035	0.050
Absent	13(50.0)	24(52.2)		2(28.6)	5(45.5)		
Present	12(46.2)	22(47.8)		2(28.6)	6(54.5)		
Not available	1(3.8)	0(0)		3(42.9)	0(0)		

Data are numbers of patients, with percentages in parentheses

^a^Data are medians, with inter-quartile range

### Feature selection and development of a radiomics model

**Fig. 3:**
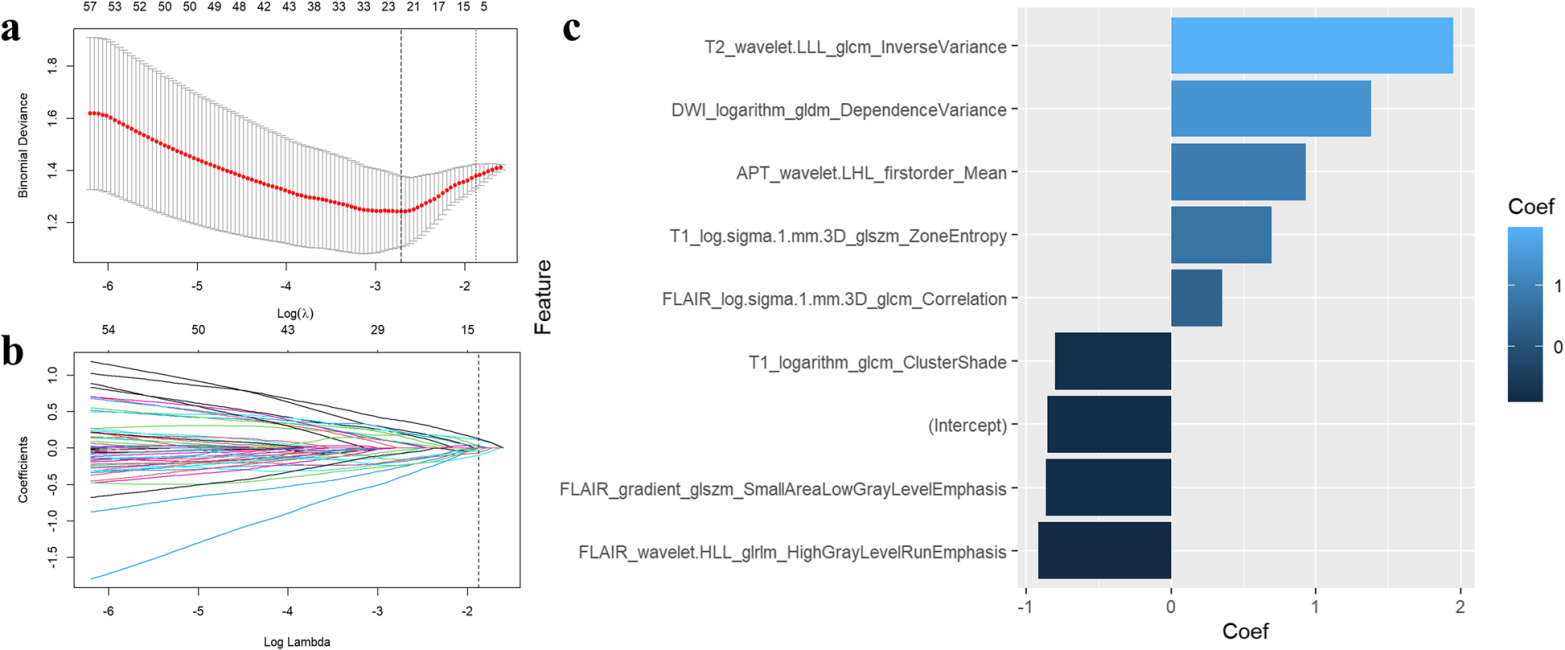
LASSO coefficient profiles of the 8454 radiomics features. A coefficient profile plot was generated versus the selected log λ value using fivefold cross-validation (**a**). Optimal parameter selection in the LASSO model via 1-standard error criterion (**b**) The coefficients of 8 most relevant radiomics features and intercept (**c**)

**Fig. 4:**
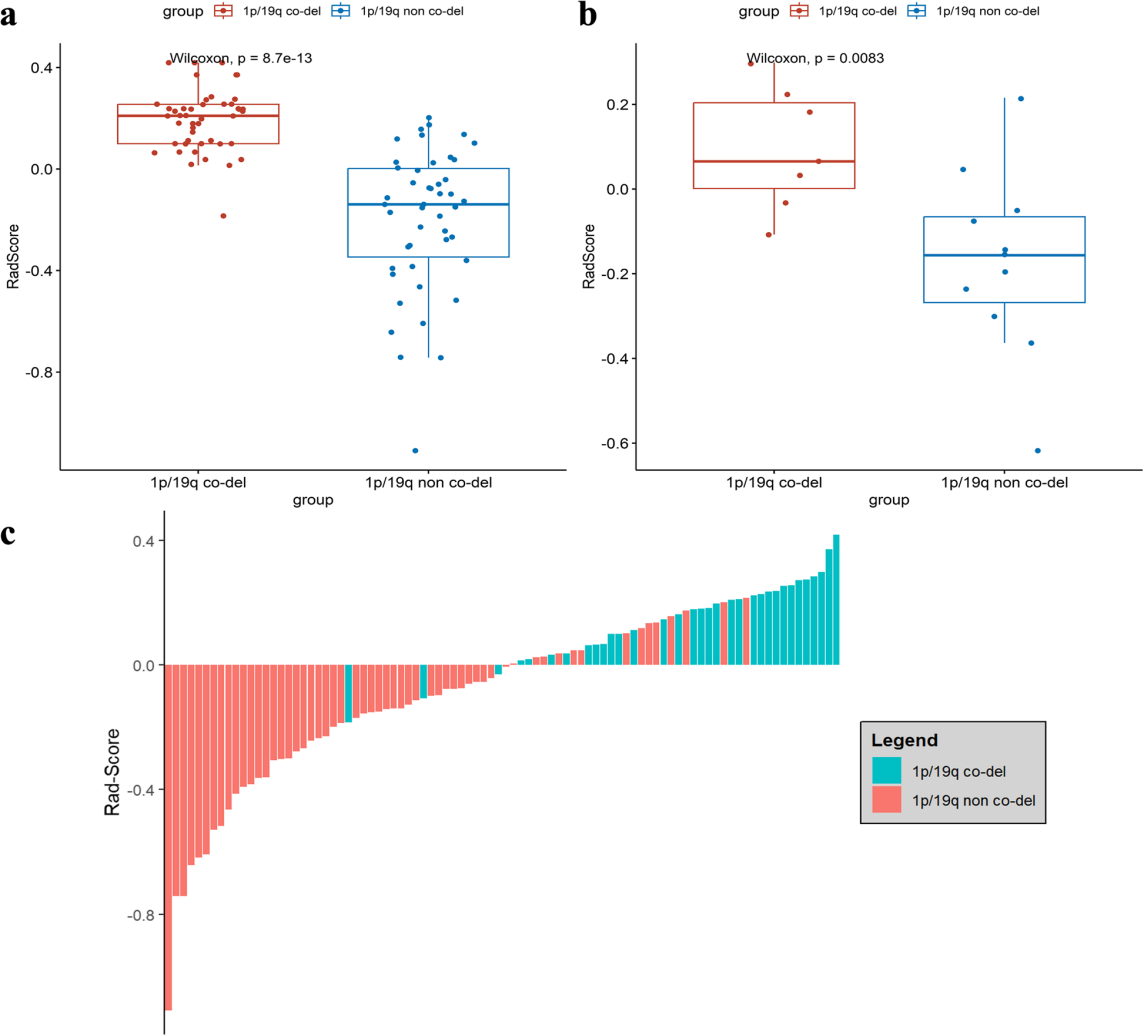
Comparation of Rad-Score between glioma patients with 1p/19q co-deleted and 1p/19q non co-deleted in training (**a**) and test sets (**b**). The bar chart of Rad-score (**c**)

Overall, 8454 radiomic features were extracted from multiparametric MRI sequences (T1, T2, FLAIR, DWI, APTw images, and ADC maps) for each patient. After application of the LASSO regression model, 8 highly relevant radiomic features were selected to construct the radiomic model (Fig. 3, a and b, Table 2) and the coefficients of the features were demonstrated in Fig. 3, c. There was a significant difference in the Rad-score between 1p/19q co-deleted and 1p/19q non co-deleted gliomas in the both training and test sets (Fig. 4 a and b). The bar chart for Rad-score can be found in Fig. 4c.

**Table 2:** Radiomics feature selection results

Variables	Radiomics feature name	Variables	Radiomics feature name
A	T1_logarithm_glcm_ClusterShade	E	FLAIR_log.sigma.1.mm.3D_glcm_Correlation
B	T1_log.sigma.1.mm.3D_glszm_ZoneEntropy	F	FLAIR_wavelet.HLL_glrlm_HighGrayLevelRunEmphasis
C	T2_wavelet.LLL_glcm_InverseVariance	G	DWI_logarithm_gldm_DependenceVariance
D	FLAIR_gradient_glszm_SmallAreaLowGrayLevelEmphasis	H	APT_wavelet.LHL_firstorder_Mean

### Classification performance and model comparison with neuroradiologist

The performance of the three models classifying the molecular subtypes of LGG in the training and test sets are shown in Table 3. The results of the DeLong test comparing the predictive performance of the three models against neuroradiologists (Reader A, B, and C) in both the training and test sets are summarized in Table 4.

**Table 3:** Diagnostic performance by different models

Model	AUC (95% CI)	Sensitivity (%)	Specificity (%)	PPV (%)	NPV (%)	Accuracy (%)
Training set (*n* = 72)						
Clinical model	0.761 (0.946–0.871)	73.7	77.4	53.8	89.1	76.4
Radiomics model	0.948 (0.909–0.987)	83.7	88.4	89.1	82.6	85.9
Combined model	0.966(0.932–0.999)	87.5	90.9	91.3	87.0	89.1
Reader A	0.804(0.723–0.886)	82.6	78.3	79.2	81.8	80.4
Reader B	0.739(0.658–0.820)	93.5	54.3	67.2	89.3	73.9
Reader C	0.674(0.588–0.760)	89.1	45.7	62.1	80.8	67.4
Test set (*n* = 18)						
Clinical model	0.766 (0.525–1)	80.0	76.9	57.1	90.9	77.8
Radiomics model	0.909 (0.770–1)	80.0	76.9	57.1	90.9	77.8
Combined model	0.896 (0.733–1)	80.0	76.9	57.1	90.9	77.8
Reader A	0.740(0.523–0.957)	57.1	90.9	80.0	76.9	77.8
Reader B	0.604(0.356–0.852)	57.1	63.6	50.0	70.0	61.1
Reader C	0.513(0.262–0.764)	57.1	45.4	40.0	62.5	50.0

*AUC* area under the curve, *CI* confidence interval, *PPV* positive predictive value, *NPV* negative predictive value

**Table 4:** *P* values in comparing AUC of Delong test

	Training set				Test set	
	Clinical model	Radiomics model	Combined model	Clinical model	Radiomics model	Combined model
Rader A	0.53	0.001	< 0.001	0.88	0.26	0.28
Rader B	0.76	< 0.001	< 0.001	0.38	0.03	0.053
Rader C	0.23	< 0.001	< 0.001	0.13	0.02	0.02

**Fig. 5:**
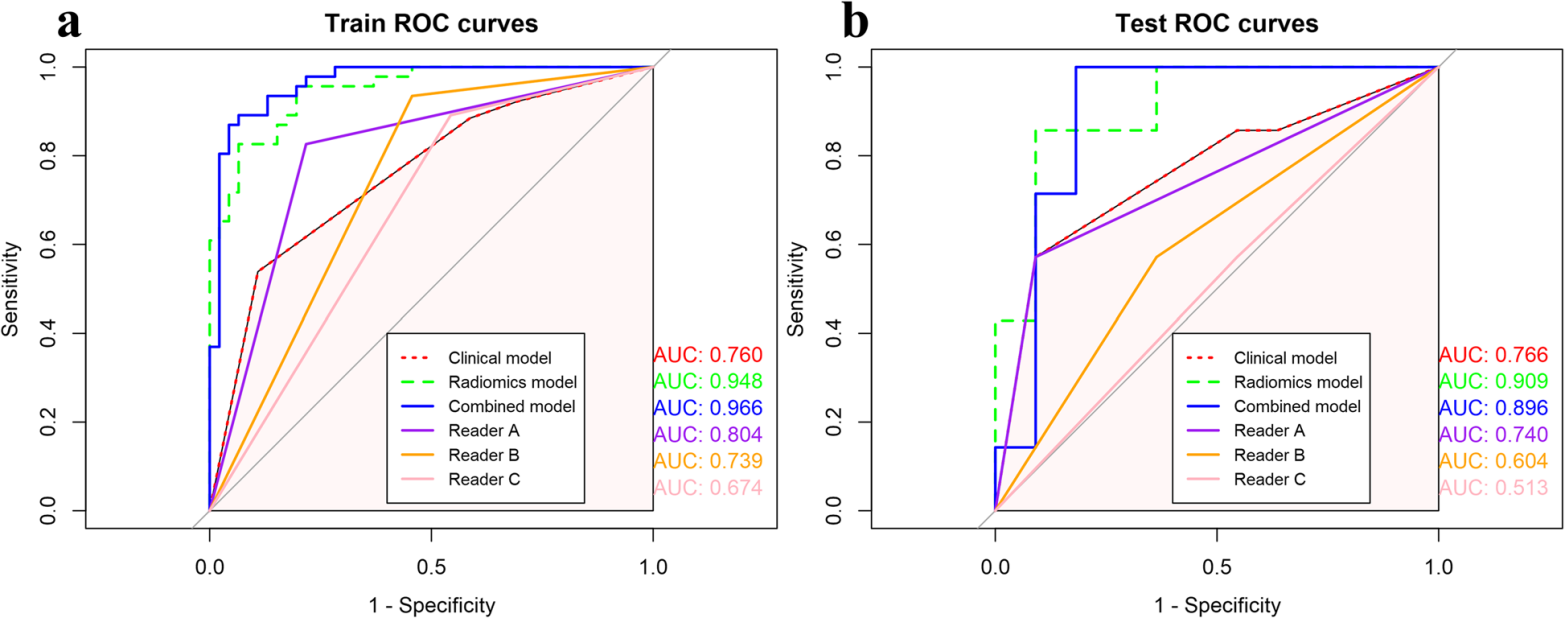
ROC curves of the models and signatures in the training (**a**) and test set (**b**)

After performing the Delong analysis, it was found that there was no statistically significant difference in predictive performance between the clinical model and the three readers (A, B, C). In the training set. Both the radiomic model and the combined model performed significantly better than the three readers. In the test set, the AUC values of the three models were higher than those of the three readers, but there was no statistically significant difference compared to the experienced neuroradiologist (Reader A). On the other hand, the radiomics model significantly outperformed the resident physicians (Reader B and C). The ROC curves for the three models and the three neuroradiologists in both the training and test sets are presented in Fig. 5.

**Fig. 6:**
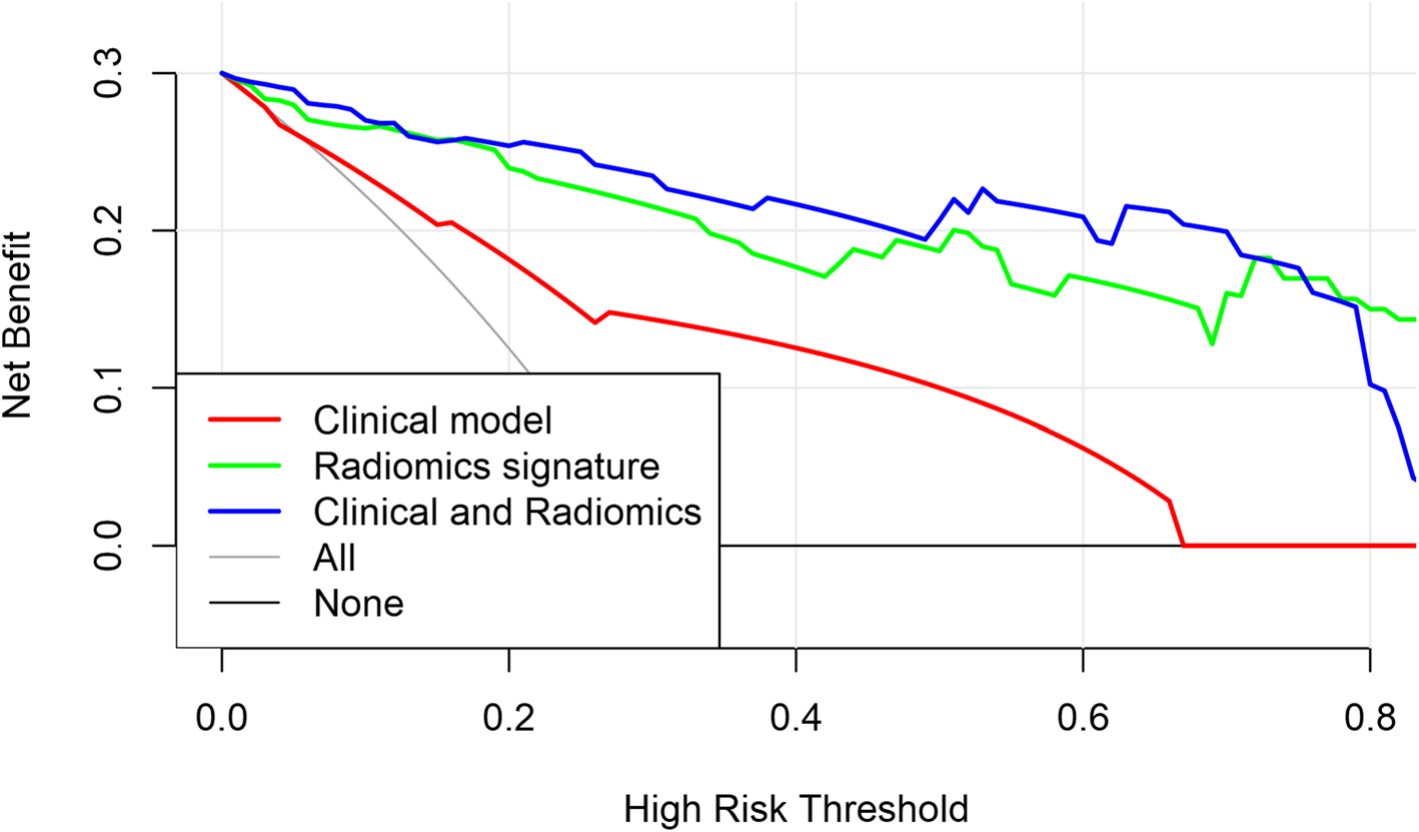
Decision curve analysis for the three models

Meanwhile, the decision curves for molecular subtype classification among the three models demonstrate that the radiomic model and combined model have a better overall net benefit compared to the clinical model (Fig. 6).

## Discussion

In this study, we developed a radiogenomics method that predicts 1p/19q co-deletion status in LGG based on APTw, DWI, conventional sequences, as well as additional MRI features. Our results showed that the radiomics model and the combined model exhibited excellent performance in distinguishing 1p/19q co-deletion status in both the training and test sets, with AUCs of 0.948 and 0.966 for the training set, and 0.909 and 0.896 for the test set, respectively. Furthermore, the predictive performance of our model was comparable to that of experienced neuroradiologist, significantly outperforming the diagnostic accuracy of resident physicians. 1p/19q co-deletion is associated with longer progression-free and overall survival, and better response to radiotherapy and chemotherapy [1, 25, 26]. For the suspected LGG patients, maximal safe surgical resection is advocated as the standard of care [27]. However, recent studies demonstrated that gross total resection was not related with prolonged survival of patients with oligodendroglioma [28, 29]. Therefore, preoperative identification of 1p/19q genotype could help with surgical planning.

There are growing number of studies using machine learning algorithms to predict molecular subtypes such as 1p/19q co-deletion and IDH mutations [7, 21, 30–33]. However, most of these studies only used conventional anatomical MR sequences because of the widespread usage. The 3D APTw and DWI in our datasets enabled us to extract molecular information from the tumor. Recently, two pioneering APTw-based radiomics studies have been undertaken, aiming to distinguish glioblastomas from gliomas and brain metastases, as well as to discriminate treatment response from tumor progression [34, 35]. Our research findings indicate that APTw-based radiomics also holds value in predicting the 1p/19q co-deletion in LGG.

In the training set, our findings indicate statistically significant differences in calcifications, tumor margin clarity, and gray matter involvement between gliomas with 1p/19q co-deletion and those without, which is consistent with prior studies. Notably, among these features, only calcifications and tumor margin clarity independently predict 1p/19q co-deletion. This may be related to the relatively small sample size of our training dataset. In the radiomics model, our results demonstrate that among the three most contributing features two are texture features from T2WI and DWI while the third is a histogram feature from APTw (Fig. 3, c). This finding could partly be explained by the fact that 1p/19q-codeleted glioma frequently had heterogeneous signal intensity on T2WI [6, 36], mixed/restricted diffusion characteristics [37], and concentration of endogenous cellular proteins in tissue, which can be reflected by radiomic features from APTw, varies across different molecular subtypes of gliomas [38].

Of all these models, the radiomics model achieved the best performance in the testing set, which means the radiomics model had strong predictive power for 1p/19q co-deletion. The clinical model demonstrates similar performance in both the training and testing sets with an AUC of 0.760 and 0.766. However, in the training set, the AUC value of the radiomics model is lower than that of the combined model. Contrastingly, in the testing set, the AUC value of the radiomics model is higher than that of the combined model. This observed difference could be attributed to the relatively smaller sample size of the testing set, indicating the need for a larger dataset evaluation to draw more conclusive results.

Our study has several limitations. First, it was based on a single-center, retrospectively collected dataset, multi-center data will be needed to allow external validation. Secondly, other medical images including perfusion-weighted imaging (PWI) and computed tomography (CT) may provide extra functional and calcified information; accordingly, we suggest that further work includes more imaging modalities to explore the performance of radiomic models. Finally, our work only focused on the prediction of the 1p/19q co-deletion genotype, the analysis of other molecular subtype, including IDH1/2, and CDKN2A/B will permit additional comprehensive understanding of the diffuse gliomas.

## Conclusions

Radiomic features from APTw, DWI and conventional MRI sequences can preoperatively and non-invasively distinguish the 1p/19q co-deletion genotype in patients with LGG. The predictive performance of radiomics model was comparable to that of experienced neuroradiologist, significantly outperforming the diagnostic accuracy of resident physicians. These findings suggest that our radiogenomics approach has the potential to become a valuable tool in clinical decision making for LGG patients.

### Supplementary Information

**Supplementary Material 1. **

## Data Availability

The datasets generated or analyzed in the current study are not publicly available because of patient privacy protection, but are available from the corresponding author upon reasonable request.

## Abbreviations

LGG: Low-grade gliomas
APTw: Amide proton transfer weighted
DWI: Diffusion weighted imaging
LASSO: Least absolute shrinkage and selection operator
Rad-score: Radiomics score
IDH: Isocitrate dehydrogenase
T1WI: T1-weighted imaging
FLAIR: Fluid-attenuated inversion recovery
T1C: Contrast-enhanced T1-weighted imaging
CEST: Chemical exchange saturation transfer
MGMT: Human O6-methylguanine DNA methyltransferase
H3K27M: Lys-27-Met mutations in histone 3 genes
DSW: Direct saturation of water
MTC: Semi-solid magnetization transfer contrast
MTR: Magnetization transfer ratio
FISH: Fluorescence in situ hybridization
LSI: Locus specific identifier
TR: Repetition time
TE: Echo time
VOI: Volume of interest
ADC: Apparent diffusion coefficient
GLCM: Gray level co-occurrence matrix
GLRLM: Gray level run length matrix
GLSZM: Gray level size zone matrix
NGTDM: Neighboring gray tone difference matrix
GLDM: Gray level dependence matrix
AUC: Area under the curve
ROC: Receiver operating characteristics
PPV: Positive predictive value
NPV: Negative predictive value
PWI: Perfusion-weighted imaging
CT: Computed tomography
