# Supplementary material for: Amide proton transfer weighted and diffusion weighted imaging based radiomics classification algorithm for predicting 1p/19q co-deletion status in low grade gliomas
Source: BMC Med Imaging. 2024 Apr 10;24:85. doi: 10.1186/s12880-024-01262-z (PMC11005152; doi:10.1186/s12880-024-01262-z)
Supplement: Supplementary file 1 — Supplementary Material 1. [file 12880_2024_1262_MOESM1_ESM.docx]

**Supplementary Data:**

| Parameters | TIWI | T2WI | FLAIR | DWI | 3D APTw |
| --- | --- | --- | --- | --- | --- |
| **TR (ms)** | 2000 | 3000 | 9000 | 3284 | 5900 |
| **TE (ms)** | 20 | 90 | 120 | 200 | 8 |
| **TI (ms)** | 800 | N/A | 2500 | N/A | N/A |
| **FA (°)** | 90 | 90 | 90 | 90 | 90 |
| **Section thickness (mm)** | 4.4 | 4.4 | 4.4 | 4.4 | 5.4 |
| **Image slice spacing (mm)** | 1 | 1 | 1 | 1 | N/A |
| **FOV (mm^2^)** | 230 × 187 | 230 × 185 | 230 × 183 | 230 × 230 | 212 × 182 |
| **Matrix** | 272 × 167 | 328 × 216 | 352 × 144 | 152 × 122 | 120 × 102 |

**Supplementary Table 1 Conventional** MR imaging parameters
